# Efficient design of a phase III trial of competing tests for personalised cancer treatment in the absence of gold standard outcome data: challenges and potential solutions

**DOI:** 10.1186/1745-6215-14-S1-O5

**Published:** 2013-11-29

**Authors:** Peter Hall, Christopher McCabe, Claire Hulme, Richard Edlin, Janet Dunn, David Cameron, Rob Stein

**Affiliations:** 1 University of Leeds, Leeds, UK; 2 University of Warwick, Warwick, UK; 3 University of Alberta, Edmonton, Canada; 4 University of Auckland, Auckland, New Zealand; 5 University College London, London, UK; 6 University of Edinburgh, Edinburgh, UK

## Introduction

The OPTIMAprelim pilot trial in early breast cancer aims to select multi-parameter tests for inclusion in a phase III trial evaluating their use for patient selection for chemotherapy. Patients are randomised between universal chemotherapy or test-directed chemotherapy. A key criterion for inclusion of a test as a comparator will be the expected value of further research into its clinical and cost-effectiveness.

## Methods

In order to conduct a Value-of-Information (VoI) Analysis a probabilistic decision model was developed to evaluate the cost-effectiveness of a personalised treatment strategy based on each of the candidate tests from a UK NHS perspective. Concordance/discordance with Oncotype DX, the gold standard test, was used to model expected sensitivity and specificity of alternative tests.

## Results

Decision analytic models of test guided cancer therapy require the explicit characterisation of test performance for different test definitions and survival conditional upon test result. In this example, modelling the required outcome data, dependent on the modelled test result, was feasible although likely represents an underestimate of the true uncertainty. It was possible to describe relative value of research on the test characteristics versus research on chemotherapy effectiveness across test-defined patient sub-groups. Modelling solutions which consider alternative test cut-points and time-dependent outcomes are presented.

## Conclusion

Characterising the uncertainty and value of information associated with test performance parameters and the expected outcomes conditional upon test results is feasible on the basis of test concordance data, even with limited current long term outcomes data.

